# Elderberry extract improves molecular markers of endothelial dysfunction linked to atherosclerosis

**DOI:** 10.1002/fsn3.3393

**Published:** 2023-05-10

**Authors:** Joseph Festa, Aamir Hussain, Amon Hackney, Unmesh Desai, Tarsem S. Sahota, Harprit Singh, Mariasole Da Boit

**Affiliations:** ^1^ Leicester School of Allied Health Sciences De Montfort University Leicester UK; ^2^ Leicester School of Pharmacy Faculty of Health and Life Sciences De Montfort University Leicester UK

**Keywords:** anthocyanins, atherosclerosis, elderberries, endothelial dysfunction, endothelial nitric oxide synthase

## Abstract

Endothelial dysfunction (ED), secondary to diminished nitric oxide (NO) production and oxidative stress, is an early subclinical marker of atherosclerosis. Reduced NO bioavailability enhances the adhesion of monocytes to endothelial cells and promotes atherosclerosis. Elderberry extract (EB) is known to contain high levels of anthocyanins which could exert vascular protective effects. Specifically, we investigated the functional capacity of EB on various markers of ED. Human umbilical vein endothelial cells (HUVEC) were pretreated with EB 50 μg/mL and stimulated with TNF‐α 10 ng/mL. Cell viability, apoptosis, oxidative stress; eNOS, Akt, Nrf2, NOX‐4, and NF‐κB at the protein level were measured. A co‐culture model was used to determine whether EB could prevent the adhesion of monocytes (THP‐1) to HUVECs. Moreover, the expression of adhesion molecules and pro‐inflammatory cytokines were also measured. It was demonstrated that EB prevented TNF‐α induced apoptosis and reactive oxygen species production in HUVECs. Additionally, EB upregulated Akt and eNOS activity, and Nrf2 expression in response to TNF‐α, whereas it decreased NOX‐4 expression and NF‐κB activity. EB prevented the adhesion of monocytes to HUVECs, as well as reduced IL‐6 and MCP‐1 levels, which was associated with inhibition of VCAM‐1 expression. Our results demonstrate that EB upregulates key cellular markers of endothelial function and ameliorates markers of ED. EB could be used as a potential nutritional aid for preventing atherosclerosis progression.

## Graphical abstract

Endothelial dysfunction (ED) is an early sub‐clinical marker of atherosclerosis. We investigated the functional capacity of elderberry (EB) on different markers of ED. Our results demonstrate that EB upregulates key cellular markers of endothelial function and ameliorates markers of ED.

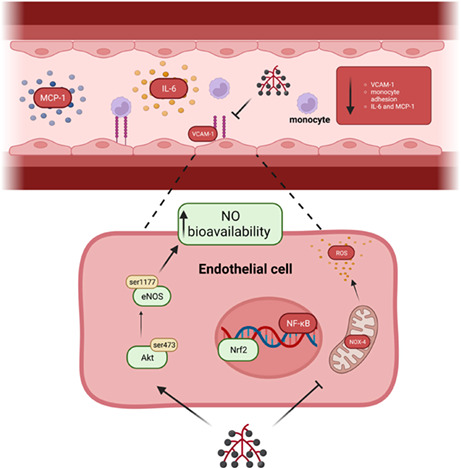

## 1 INTRODUCTION

Cardiovascular disease (CVD) remains the most common cause of death globally (Roth et al., 2018). One common marker for most CVD is the early development of endothelial dysfunction (ED) as a disruption in nitric oxide (NO) bioavailability occurs (Mudau et al., 2012; Münzel & Daiber, 2018). NO is the predominant vasodilator found within the vasculature and prevents the adhesion of leukocytes to the vascular endothelium and leukocyte migration into the vascular wall (Förstermann et al., 2017). The phosphatidylinositol‐3 kinase (PI3K)/Akt pathway is a downstream regulator of endothelial nitric oxide synthase (eNOS), an enzyme that generates the production of NO in endothelial cells (EC) (Dimmeler et al., 1999; Münzel & Daiber, 2018). Furthermore, the enhanced production of reactive oxygen species (ROS) that exceeds the capacity of cellular antioxidants termed “oxidative stress” is also a major contributing factor to ED (Mudau et al., 2012). NOX family of NADPH oxidases are major sources of ROS implicated in the production of the superoxide that combines with NO to form peroxynitrite which is responsible for eNOS uncoupling and impairment of NO bioavailability (Figueira et al., 2013; Lee et al., 2013). This is part of the early stages of vascular inflammation, which ultimately contributes to the progression of ED (Mudau et al., 2012).

During the later stages of ED, oxidative stress causes a proinflammatory state on the vessel wall, which is regulated by the transcription factor nuclear factor kappa B (NF‐κB) (Förstermann et al., 2017). NF‐κB is usually found inactive in the cytoplasm and its activation is initiated by the signal‐induced degradation of IκB proteins, with consequent release of NF‐κB into the nucleus where it coordinates the inflammatory process (Gilmore & Herscovitch, 2006). NF‐κB activity mediates the expression of cell adhesion molecules (CAM) and their adhesion to the vessel wall (Griendling & FitzGerald, 2003; Taniyama & Griendling, 2003). Reactive oxygen species can also directly upregulate CAM including vascular adhesion molecule‐1 (VCAM‐1) and intracellular adhesion molecule‐1 (ICAM‐1) (Griendling & FitzGerald, 2003; Taniyama & Griendling, 2003). Overexpression of VCAM‐1 and ICAM‐1 on the surface of EC interact with antigens on the surface of leukocytes, which causes the adhesion of monocytes (Virdis et al., 2014). MCP‐1 a proinflammatory cytokine is also involved with the adhesion process (Dai et al., 2004). Once monocytes are recruited to the site of inflammation interleukin 6 (IL‐6) is secreted which further perpetuates inflammation with further progression of atherosclerosis (Albertini et al., 2005; Amin et al., 2015).

Many studies have shown the potential benefits of berry anthocyanins to reduce the risk of CVD mortality by improving endothelial function (Festa et al., 2021; Kimble et al., 2019). Polyphenols and specifically berry anthocyanins can directly upregulate the nuclear factor erythroid 2‐related factor 2 (Nrf2) a transcription factor, responsible for both the constitutive and inducible expression of the antioxidant response element genes (Festa et al., 2021). Anthocyanins modulate the adhesion of monocytes to EC by decreasing inflammatory markers, including pro‐inflammatory cytokines and/or adhesion molecules (Krga et al., 2018). Elderberries (EB) in particular, are considered one of the richest sources of anthocyanins and phenolic compounds, which contribute to the high antioxidant activity of these berries (Festa et al., 2022a; Ozgen et al., 2010; Wu et al., 2004). Black elderberry is reportedly one of the richest sources of cyanidin‐3‐glucoside (C3G) (794 mg/100 g) (Duymuş et al., 2014; Pérez‐Jiménez et al., 2010). Its composition is mainly made up of cyanidin‐3‐sambucoside and C3G which account for 90% of its total anthocyanin content and has demonstrated better absorption compared to other berries (Prior et al., 2002). EB has displayed protective effects against oxidative stressors in EC and the ability to modulate inflammation activity in macrophages and dendritic cells (Thanh et al., 2017; Youdim et al., 2000). Moreover, in vivo studies demonstrate improved atherosclerotic plaque stability and high‐density lipoprotein (HDL) function in apoE−/− mice as well as lowering insulin resistance in type 2 diabetic rats after chronic EB supplementation (Millar et al., 2018; Salvador et al., 2017). However, despite the richness of anthocyanins, little has been explored on the potential protective effects of EB on ED, especially related to the initial stages of atherosclerosis. This suggests the mechanisms of action are worth investigating to understand the protective properties of EB (Bell & Gochenaur, 2006). Therefore, the main aim of this study was to investigate the functional and mechanistic capacity of EB on ED markers associated with atherosclerosis.

## 2 EXPERIMENTAL SECTION

### 2.1 Materials

Human umbilical vein endothelial cells (HUVECs) Gibco Invitrogen (cat: C‐015‐10C); Human large vessel endothelial basal media Gibco Invitrogen (cat: M200500); low serum growth supplement (LSGS) Gibco Invitrogen (cat: S‐003‐10); phosphate buffer saline (PBS) 7.4 Gibco Invitrogen (cat: 10010023); penicillin–streptomycin (10,000 U/mL; cat: 15140122); trypsin‐EDTA 10× (cat: T4174); fetal calf serum (FCS) (cat: C8056). Elderberry: (Berrypharma Schwarzes holunder extract 13%, IPRONA); celltracker™ green CMFDA (5‐chloromethylfluorescein diacetate, Invitrogen); RPMI‐1640 cell media (Thermofisher Scientific, cat. 11875093); IL‐6 (Human IL‐6 Duo‐set ELISA, Cat. DY206, Lot. P154822); MCP‐1 (Human CC12/MCP‐1 Duo‐set ELISA, Cat. DY279, Lot. P286953); Wortmannin (Novex™ Wortmannin, Fisher Scientific, cat. 10671104); H_2_O_2_ (100 mL) Sigma‐Aldrich (cat: 216763). Bovine serum albumin (BSA) Sigma (cat: A7906); MTT (98%) Sigma‐Aldrich (cat: M2128‐1G); DMSO (99.6%) (cat: 471267); tissue culture plates, 96‐well flat bottom with low evaporation lid, corning Ltd (cat: 353072); Nunclon delta surface 6‐well plate (cat: 140675); Nunclon delta surface 12‐well plate (cat: 150628). Western blot antibodies: eNOS AF950, p‐eNOS MAB9028, actin PA1‐46296, akt‐p AF887, akt 281046, NF‐κB MAB5078, NF‐κB p‐p65 72261, Nrf2‐AF3926, NOX‐4 NB110 – 58849SS were all purchased from R&D systems, UK. Flow cytometry materials: FITC annexin V A13199, VCAM‐1 12‐1069‐42, ICAM‐1 12‐0549‐41, Rat IgG1 kappa isotype control 17‐4301‐81 and mouse IgG1 kappa isotype control 25‐4714‐52 were all purchased from Invitrogen, UK.

### 2.2 Cell culture and stimulation

Primary HUVECs were maintained in Human large vessel endothelial basal media supplemented with LSGS 2%, FCS 10%, and penicillin–streptomycin 1%. Cells were incubated at 37°C in 5% CO_2_ to promote growth and maintain a constant pH. HUVECs were sub‐cultured every 48‐h to maintain intensity and for experiments, were used at passage three. Prior to stimulation, cells were serum starved for 1‐h. EB extract was dissolved in PBS at 5 mg/mL and diluted in EM‐200 media to a working concentration of 50 μg/mL. A concentration of 10 ng/mL was used throughout the study for TNF‐α based on previous studies (Festa et al., 2022b; In‐Ho et al., 2018; Iwashima et al., 2019; Kuntz et al., 2015). PBS (1:10 dilution) and H_2_O_2_ (1:1000) solutions were used as negative and positive controls, respectively.

### 2.3 Cell viability assay

Cell viability was determined by using the dimethylthiazol‐diphenyltetrazolium bromide (MTT) assay (Borra et al., 2009). Cells were seeded at 3.5 × 10^3^ per well and stimulated with EB for 24‐h +/− in the presence of TNF‐α for an additional 24‐h. After treatments, cells were washed with PBS, and MTT 5 mg/mL was added and incubated for 4‐h. DMSO was added to each well to lyse the cells and solubilize the purple formazan product and the absorbance of each well was measured at 560 nM using a plate reader. The cell viability was determined with the following formula (Huang et al., 2020):

$$\begin{array}{c} \text{Cell viability}\;\left(\%\right)\\ =\frac{\text{Sample group}\;\mathrm{OD}\;\text{value}-\text{blank group}\;\mathrm{OD}\;\text{value}\;}{\text{Control group}\;\mathrm{OD}\;\text{value}-\text{blank group}\;\mathrm{OD}\;\text{value}}\times 100\% \end{array}$$

### 2.4 Immunoblot

A time‐course experiment was conducted in HUVECs to monitor changes in activity of Akt and eNOS after the stimulation of EB at various time points including 0.5, 1, 4, 8, and 24‐h. Furthermore, HUVECS were pretreated with EB for 1‐h before being stimulated with TNF‐α for 4‐h. Cells were then lysed in RIPA buffer and the protein supernatant was collected by centrifugation at 13,000 × *g* at 4°C. Proteins were denatured by the addition of 4× laemmli sample buffer containing beta‐mercaptoethanol (100 mM) and heated at 95°C for 5 min before being resolved by 8% SDS‐page. Separated proteins were then transferred to a nitrocellulose membrane for antibody probing. Following blocking with 5% nonfat dry milk in TBST (Tris buffer saline solution in 0.1% Triton‐x100), membranes were probed separately with primary antibodies including p‐Akt (Ser^473^), p‐eNOS (ser^1177^), total Akt, eNOS, NF‐κB p‐p65, NF‐κB, NADPH oxidase isoform 4 (NOX‐4), and Nuclear factor erythroid 2‐related factor 2 (Nrf2). Membranes were washed three times in TBST and incubated with the relevant secondary antibody labeled with horseradish peroxidase. A substrate for the horseradish peroxidase enzyme was applied to the membrane, and the light emitted was captured via chemiluminescence. Optical densities of bands on immunoblots were quantified using ImageStudioLite and bands were normalized by using β‐actin as a loading control. All data were expressed as a fold change to either negative control or TNF‐α as stated.

### 2.5 Flow cytometry of intracellular ROS and apoptosis

Reactive oxygen species and apoptosis were analyzed using BD accuri C6 flow cytometry. HUVECs were maintained as described above and were seeded in a 12‐well plate at a density of 0.2 × 10^6^/mL and stimulated with EB for 24‐h +/− in the presence TNF‐α for an additional 24‐h. After stimulation, adherent cells were trypsinized with 0.5% EDTA for approximately 2 min, washed twice with cold PBS, and then centrifuged for 5 min at 600 *g*. For ROS analysis 5 μM of DCFHDA was added to cell suspension and incubated at 37°C for 30 min. Afterward, the cells were fixed with 2% FBS and subjected to flow cytometry to measure the levels of ROS. A separate set of adherent cells, following the same procedure were imaged using the eVOS XL core imaging system. For the apoptosis assay cells were resuspended at a density of 1 × 10^6^ in 400 μL 1× binding buffer, and 5 μL of annexin V‐FITC (Fisher Scientific). Cells were incubated in the dark for 15 min at 4°C and propidium iodide was added for the last 5 min. Cells were washed with 1 mL of PBS, centrifuged at 600 *g* for 5 min, and resuspended in 1× binding buffer for flow cytometry analysis.

### 2.6 Flow cytometry of adhesion molecules

The expression of cell adhesion molecules, ICAM‐1 (CD54) and VCAM‐1 (CD106) were analyzed using flow cytometry. HUVECs were maintained as described above and were seeded in a 12‐well plate at a density of 0.2 × 10^6^/mL and stimulated with EB 50 μg/mL for 24‐h +/− in the presence of TNF‐α 10 ng/mL for an additional 24‐h. Following treatments, HUVECs were detached with a nonenzymatic solution of Versene, collected, washed with 1 mL of PBS, centrifuged, and resuspended in 50 μL of PBS. Samples were then single stained with 2.5 μL of conjugated antibodies, and immunoglobin isotypes labeled with PE or just cells were used as a negative control. After 30 min of incubation at 4°C in the dark, the cells were washed with 1 mL of PBS, centrifuged at 600 *g* for 5 min, and resuspended in 350 μL 2% FBS in PBS for the analysis.

### 2.7 Monocyte adhesion assay

THP‐1 monocytes were cultured in complete RPMI cell media supplemented with 10% FBS at 37°C in 5% CO_2_. Monocyte adhesion assay was carried out as previously done by others with slight modifications (Del Bo et al., 2019; Marino et al., 2020). Briefly, HUVECs were seeded 2 × 10^4^ per well in a 96‐well black plate and stimulated with EB 50 μg/mL for 24‐h +/− in the presence of TNF‐α 10 ng/mL for an additional 24‐h. After stimulation, THP‐1 cells (2 × 10^6^) were resuspended in 1 mL serum‐free RPMI cell media and labeled with 2 μM celltracker™ green CMFDA for 1‐h. After labeling, THP‐1 cells were rinsed twice with PBS and resuspended in HUVECs media at a density of 2 × 10^5^ mL^−1^ and were added to the treated cells for an additional 45 min. At this stage the supernatant was removed, centrifuged at 600 *g* for 5 min, and stored at −80°C for ELISA analysis. Cells were rinsed twice with Hank's Balanced Salt Solution (HBSS) and the fluorescence (excitation: 485 nm, emission: 538 nm) was measured in a Promega GloMax discovery system fluorescence spectrophotometer. Monocyte adhesion was calculated as a ratio of counted monocytes to HUVECs and expressed in comparison to TNF‐α, normalized to 100%.

### 2.8 Determination of cytokines released upon TNF‐α stimulation

ELISA‐based methods were used to determine the concentration of cytokines including IL‐6 and MCP‐1 secreted into culture supernatant after TNF‐α stimulations. The analysis was done according to the manufacturer's protocols. Absorbance was measured at 405 nm using a microplate reader. The reported values represent the average of triplicate wells.

### 2.9 Data analysis

Statistical analysis was performed using GraphPad Prism version 9 (USA). One‐way ANOVA or independent *t*‐test was performed to determine statistical differences between different conditions. A Bonferroni adjustment for multiple comparisons was applied. If Mauchly's test of sphericity was violated, Greenhouse–Geisser corrected values were used. Data are represented as the mean ± standard error of the mean (SEM) with significance taken as *p ≤ .05, **p ≤ .01, and ***p ≤ .001. Shapiro–Wilk test was used to assess the distribution of the data for every data set.

## 3 RESULTS

### 3.1 EB maintains cell viability and prevents TNF‐α induced apoptosis

**FIGURE 1:**
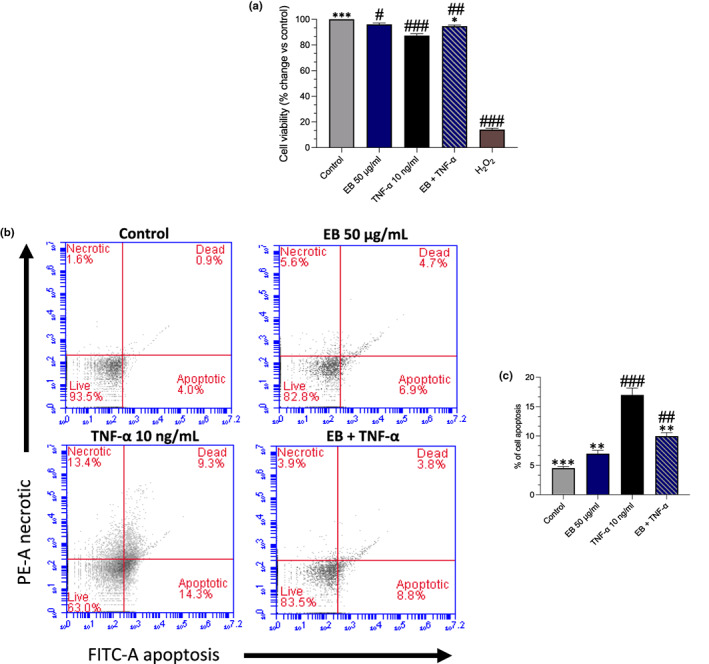
Effect of EB on cell viability and apoptosis. HUVECs were pretreated with Elderberry (EB) 50 μg/mL for 24 h and stimulated with TNF‐α for an additional 24 h. (a) MTT assay was used to assess cell viability, data presented as percentage change vs. control of mean values ± SEM. (b) Flow cytometry analysis was performed to detect the apoptotic cell population (c) Flow cytometry data presented as percentage of cell apoptosis, mean values ± SEM from *n* = 3. Significant value was set at #*p* < .05, ##*p* < .01, ###*p* < .001 vs. control; **p < .01, ***p < .001 compared with TNF‐α.

MTT assay was used to assess the viability of EC after the incubation of EB. Cells treated with the positive control (H_2_O_2_) decreased cell viability versus negative control (−86%, *p* < .0001). Additionally, TNF‐α 10 ng/mL reduced cell viability markedly versus negative control (−13%, *p* = .0010). However, EB 50 μg/mL alone did not reduce cell viability within the cells, and improved viability in the presence of TNF‐α (*p* = .0125; Figure 1a). The functional capacity of EB in the presence of TNF‐α was assessed as TNF‐α has previously been demonstrated to increase EC apoptosis (Chen et al., 2020). We detected that TNF‐α induced apoptosis over a 24‐h period (+12%, *p* = .0005) vs control, with EB suppressing TNF‐α induced apoptosis (−7%, *p* = .0056) versus TNF‐α (Figure 1b,c).

### 3.2 EB activates the Akt‐eNOS pathway

**FIGURE 2:**
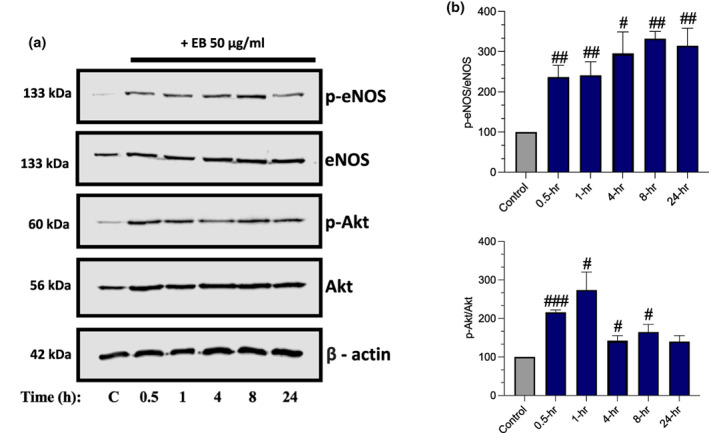
Elderberries (EB) increases Akt and eNOS activity in endothelial cells. (a) HUVECs were treated with or without EB (50 μg/mL) for the specified time (h). Cell lysates were subjected to immunoblotting and probed for phosphorylated eNOS (p‐eNOS), total eNOS, phosphorylated Akt (p‐Akt), and total Akt followed by β – actin which was used as loading control. (b) Respected graphs for eNOS and Akt. Data are presented as mean and standard error of the mean (SEM) (*n* = 3). Significant value was set at #*p* < .05, ##*p* < .01, ###*p* < 0.001 vs. control.

Western blots were used to determine whether EB can increase the activity of the Akt‐eNOS signaling pathway. It was demonstrated that EB can induce Akt and eNOS activity in a time‐dependent manner (Figure 2). Akt phosphorylation peaked at 1 h (2‐fold, *p* = .0204), whereas eNOS activity peaked at 8‐h (3‐fold, *p* = .002).

**FIGURE 3:**
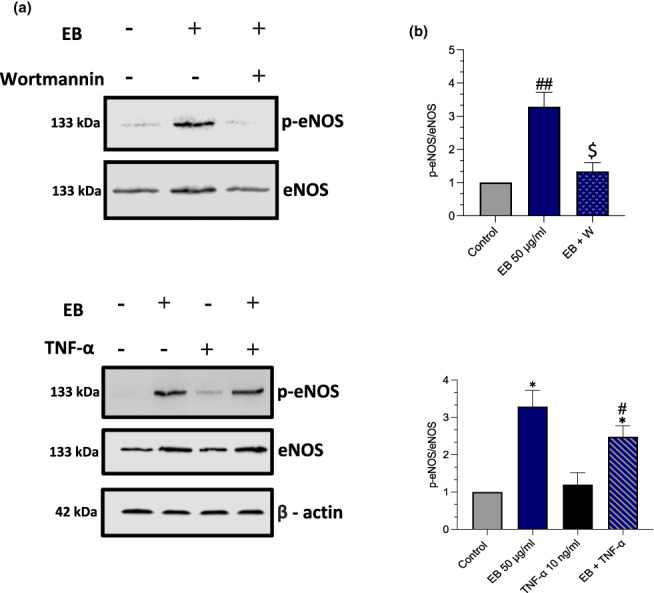
Elderberries (EB) prevents eNOS uncoupling via Akt pathway (a) HUVECs pretreated with wortmannin (W) (30 nM) for 30 min and stimulated with EB (50 μg/mL) for 1 h. (b) HUVECs were pretreated with EB 50 μg/mL for 1 h and stimulated with TNF‐α for an additional 4 h. eNOS activity was probed using western blots with its representative graph. Data are presented as mean and standard error of the mean (SEM) (*n* = 3). Significant value was set at #*p* < .05, ##*p* < .01; *p < .05, compared with TNF‐α; $*p* < .05 compared with EB 50 μg/mL.

To confirm if EB induced eNOS via the Akt pathway, HUVECs were preincubated with wortmannin (inhibitor of PI3 kinase/Akt) for 30 min (30 nM) before stimulating with EB for 1‐h (Figure 3a). Wortmannin inhibited EB‐induced eNOS activity (−1.951‐fold, *p* = .0185). After determining the capabilities of EB to induce the Akt‐eNOS pathway, we then explored its effect in the presence of TNF‐α. TNF‐α suppressed eNOS activity after a 4‐h incubation period (Figure 3b). However, pretreatment with EB for 1‐h prevented the inhibitory effect of TNF‐α and increased eNOS activation (1.2‐fold, *p* = .0423).

### 3.3 Elderberry extract protects endothelial cells against TNF‐α induced oxidative stress

**FIGURE 4:**
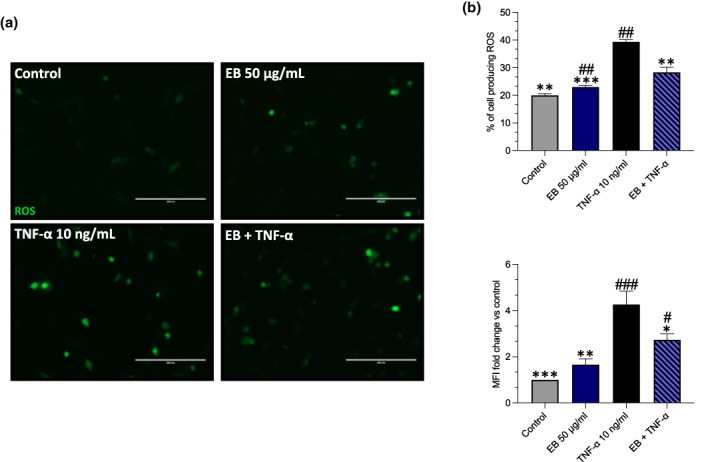
Elderberries (EB) suppresses TNF‐induced ROS in EC. Cells pretreated with EB 50 μg/mL for 24 h before being stimulated with TNF‐α for an additional 24 h. (a) ROS assay was measured using DCFHDA assay, images were taken at using the eVOS XL core imaging system, and scale bars set at 200 μm. (b) For quantification purposes, ROS was measured as % of cells producing ROS and mean fluorescent intensity (MFI) using flow cytometry and includes the representative graphs. Data are presented as mean and standard error of the mean (SEM) (*n* = 3). Significant value was set at #*p* < .05, ##*p* < .01, ###*p* < .001 vs. control; *p < .05, **p < .01, ***p < .001 compared with TNF‐α.

**FIGURE 5:**
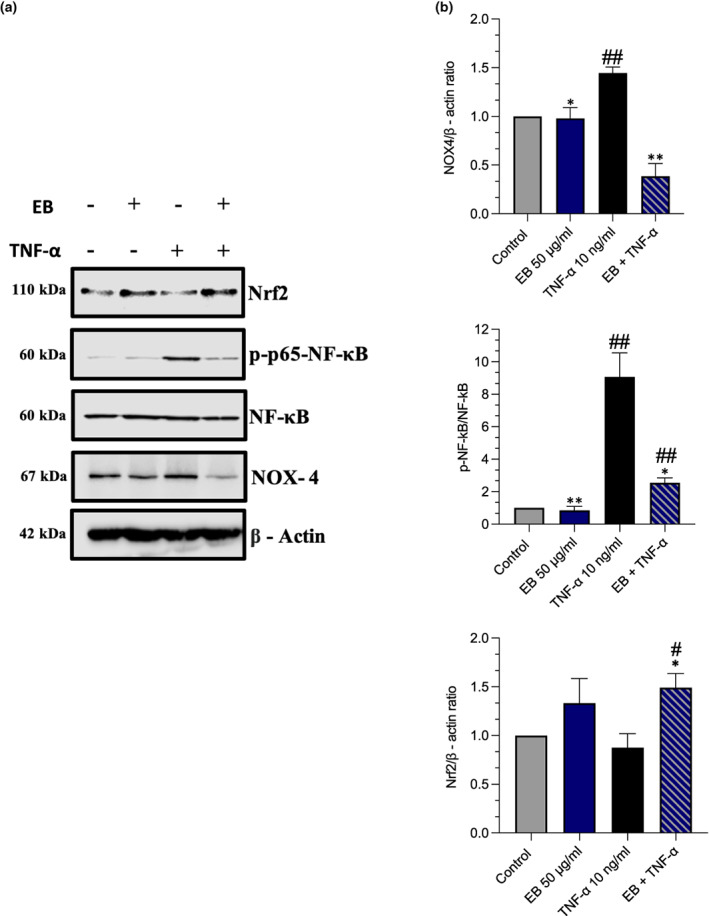
Effects of EB on NOX‐4, NF‐κB and Nrf2. (a) HUVECs were pretreated with EB 50 μg/mL for 1 h and stimulated with TNF‐α for an additional 4 h. Cell lysates were subjected to immunoblotting and probed for NF‐kB activity, NOX‐4, and Nrf2 expression (b) Includes representative graphs. Data are presented as mean and standard error of the mean (SEM) (*n* = 3). Significant value was set at #*p* < .05, ##*p* < .01, ###*p* < .001 vs. control; *p < .05, **p < .01, ***p < .001 compared with TNF‐α.

The prevention of TNF‐α induced apoptosis pretreated with EB was further explored by measuring ROS levels in HUVECs, as it is established that TNF‐α induces apoptosis in HUVECs via the production of ROS (Chen et al., 2020). Reactive oxygen species production was low in the control, with only 20% of cells producing ROS, as well as in the EB cell population (23%). An increase in ROS production and mean fluorescent intensity (MFI) was found after TNF‐α of stimulation vs control (+19%, *p* < .0001) and (+3.267‐fold, *p* = .0004) respectively (Figure 4). However, EB suppressed TNF‐α induced ROS production (−11%, *p* = .0051) and (−1.527‐fold, *p* = .0306). To explain the mechanisms by which EB was able to mitigate TNF‐α induced ROS production, we explored NOX‐4 expression. Western blotting analysis showed that intracellular protein expression levels of NOX‐4 were low in the control cells but were strongly upregulated when exposed to TNF‐α (0.4467‐fold, *p* = .0018), however, this was suppressed in the presence of EB (0.3867, *p* = .0018; Figure 5).

The expression of Nrf2 and NF‐κB activity was explored as they are both key pathways regulating the balance between cellular redox status and response to stress and inflammation (Li et al., 2008). TNF‐α caused an increase in NF‐κB activity (8‐fold, *p* = .0054). However, pretreatment with EB caused a reduction in NF‐κB activity (6.5‐fold, *p* = .0123). Interestingly we did not see any reductions in Nrf2 expression after treatment with TNF versus control (*p* = .4439). Whilst EC treated with EB alone did not increase Nrf2 expression (*p* = .2566), EC pretreated with EB in the presence of TNF‐α increased Nrf2 levels (0.6133, *p* = .0391).

### 3.4 EB reduces monocyte adhesion by suppressing VCAM‐1 but not ICAM‐1 in HUVECS

**FIGURE 6:**
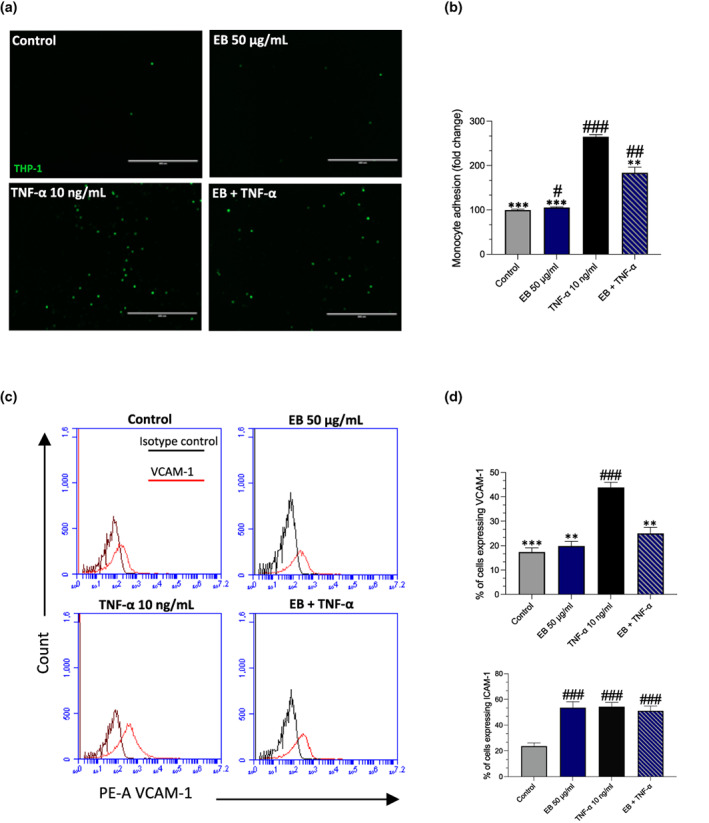
Elderberries (EB) reduces monocyte adhesion and VCAM‐1 expression. Cells pretreated with EB 50 μg/mL for 24 h before being stimulated with TNF‐α for an additional 24 h. (a) Monocyte adhesion assay images were taken using the eVOS XL core imaging system, scale bars set at 400 μm. (b) For quantification purposes, monocyte adhesion was measured using microplate reader and includes the representative graphs (c) Histogram overlays were used to compare unstained vs. stained samples. Graphs for flow cytometry VCAM (d) VCAM‐1 and ICAM‐1 representative graphs. Data are presented as mean and standard error of the mean (SEM) (*n* = 3). Significant value was set at #*p* < .05, ##*p* < .01, ###*p* < .001 vs. control; *p < .05, **p < .01, ***p < .001 compared with TNF‐α.

**FIGURE 7:**
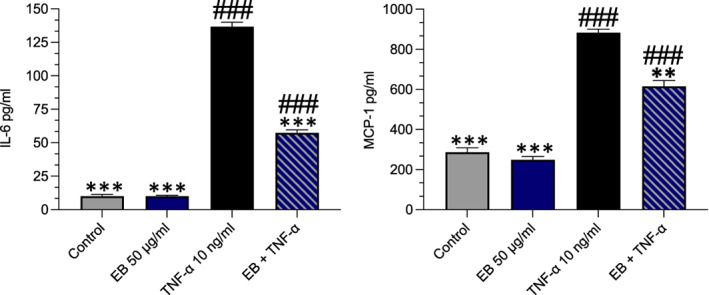
Elderberries (EB) suppresses pro‐inflammatory cytokines. Cells were pretreated with EB 50 μg/mL for 24 h before being stimulated with TNF‐α for an additional 24 h. Representative graphs for cytokines IL‐6 and MCP‐1. Data are presented as mean and standard error of the mean (SEM) (*n* = 3). Significant value was set at ###*p* < .001 vs. control; *p < .05, **p < .01, ***p < .001 compared with TNF‐α.

NF‐κB activation is a critical regulator of CAMs including ICAM‐1 and VCAM‐1 which causes the adhesion of monocytes to endothelial cells during endothelial dysfunction (Mestas & Ley, 2008). Over a 24‐h period, TNF‐α increased both VCAM‐1 expression (+26%, *p* = .0007; Figure 6) and ICAM‐1 expression (+30%, *p* < .0001). Pretreatment of EC with EB did not reduce ICAM‐1 expression although it did reduce VCAM‐1 expression (−19%, *p* = .0046). We, therefore, used an in vitro co‐culture model to investigate if EB can reduce the adhesion of monocytes to EC. EC was incubated with TNF‐α for a 24‐h period before being cultured with THP‐1 monocytes for a 45‐min period. TNF‐α increased the adhesion of monocytes (+165%, *p* < .0001) over a 24‐h period (Figure 7). However, pretreatment with EB for 24‐h prior to TNF‐α reduced the adhesion of monocytes (−81%, *p* = .0033).

### 3.5 EB suppresses the production of pro‐inflammatory cytokines IL‐6 and MCP‐1

As mentioned within the methods cell supernatant was removed before adding monocytes and post‐incubation with monocytes. We explored MCP‐1 as it has an essential role in the adhesion of monocytes (Gerszten et al., 1999). TNF‐α increased MCP‐1 in EC supernatant over a 24‐h period vs control (*p* < .001, 883 vs. 286 ng). EB suppressed this (*p* = .0013). IL‐6 is also a key inflammatory cytokine that has a major role in the inflammatory process (Collins et al., 1995). TNF‐α increased IL‐6 vs control (10 vs. 136, *p* < .001, respectively). This was suppressed within the presence of EB (136 vs. 57.43, *p* < .001).

## 4 DISCUSSION

Elderberry (EB) extract is rich in anthocyanins and phenolic compounds, specifically C3G which has been previously demonstrated to improve endothelial function. Within this study, we demonstrate the positive impact that EB has on different molecular markers of ED. We demonstrate that EB increases eNOS activation via the Akt pathway which improves cell survival, increases antioxidant defenses through Nrf2, and inhibits NOX‐4 upregulation and NF‐κB activity. In a functional model of the initial stages of atherosclerosis, EB prevents the adhesion of monocytes to EC by suppressing VCAM‐1 expression and the reduction of pro‐inflammatory cytokines. The beneficial effects shown by EB treatment could be used to promote endothelial function and positively contribute to hinder the atherosclerosis process.

Numerous studies have shown that NO bioavailability contributes to the cardiovascular health benefits associated with the consumption of polyphenol‐rich products including red wine, green tea, berries, and cocoa, although limited studies have explored EB (Fraga et al., 2011; Ndiaye et al., 2005). In our findings, we demonstrate that EB at 50 μg/mL increases Akt and eNOS activity in a time‐dependent manner over a 24‐h period with Akt peaking at 1‐h whereas eNOS peaked at 8‐h (Figure 2b). Our HPLC analysis detected C3G in our extract which could have contributed to our findings (Figure S1). Moreover, in healthy men, the consumption of 240 g of blueberries increased flow‐mediated dilation, which was closely linked to the increase in phenolic metabolites found in plasma at 1–2 h and 6‐h post‐consumption (Rodriguez‐Mateos et al., 2013). Indicating that phenolic metabolites derived from EB could have played a role in the function of the extract as seen in our eNOS data (de Ferrars, Czank, et al., 2014; Fang, 2014; Festa, et al 2023). Additionally, 12 weeks of EB supplementation demonstrated in post‐menopausal women that anthocyanin metabolites reached higher plasma concentrations than its original anthocyanin form (de Ferrars, Cassidy, et al., 2014).

For berries to induce eNOS over this period suggests that it is likely via calcium‐independent pathway, including the Akt pathway which is related to cell survival (Luzak et al., 2014; Schini‐Kerth et al., 2010). Similarly, EC stimulated for 20‐h with 600 μg/mL red wine polyphenol extract led to approximately 2.1‐fold increase in eNOS protein levels and approximately 2‐fold in NO production (Leikert et al., 2002). To confirm this, we pretreated EC with PI3‐kinase inhibitor wortmannin 30 nM for 30 min before stimulating with EB (Figure 3a). We found that wortmannin decreased eNOS activity, suggesting that EB regulates eNOS via an Akt pathway as previously mentioned, which may also explain the maintained cell viability and the prevention of TNF‐α induced apoptosis that was observed in the study (Figure 1).

During the early stages of ED eNOS uncoupling occurs, and NF‐κB has been demonstrated to contribute to this (Lee, Kim, Kwak, et al., 2014). In our study, TNF‐α at 10 ng/mL decreased eNOS activity and increased NF‐κB (eightfold) after 4‐h stimulation. Interestingly, our data demonstrate that EB prevents eNOS uncoupling by increasing eNOS activity in the presence of TNF‐α (Figure 3) and inhibits NF‐κB phosphorylation (fourfold, Figure 4). One study found that blueberries, blackcurrants, and blackberries were all able to decrease interleukin 1β and TNF‐α individually, which attenuated the lipopolysaccharide‐induced nuclear factor NF‐κB p65 activity, comparable with our findings (Lee, Kim, Yang, et al., 2014). It is likely that anthocyanins present in berry extracts elicit anti‐inflammatory properties by inactivation of proteasomal degradation of IκB‐α which causes the dephosphorylation of NF‐κB (Duarte et al., 2018; Huang et al., 2014). In rats with hyperlipidemia, the supplementation of sea buckthorn berries enhances the expression of eNOS as well as the activity of endogenous antioxidant enzymes which suppresses TNF‐α (Yang et al., 2016). It is likely that EB directly suppresses TNF‐α production, increases eNOS activity and deactivates NF‐κB.

To further explore how EB prevents eNOS uncoupling we used a functional assay (DCFHDA) to explore if EB directly inhibits ROS production which is known to cause eNOS uncoupling (Förstermann & Münzel, 2006). TNF‐α was able to directly increase ROS in EC, however, pretreatment with EB was able to directly scavenge TNF‐α ROS (Figure 4). These findings demonstrate that EB has powerful antioxidant capabilities (Duymuş et al., 2014). Furthermore, it is worth noting that EB was able to reduce NOX‐4 expression, which is a major factor of ROS production within EC (Huang et al., 2018). This may explain the prevention of eNOS uncoupling as directly inhibiting NOX‐4 reduces the scavenging of NO by superoxide anion, resulting in improved NO bioavailability. In EC isolated from db/db mice fed strawberries, suppressed NOX‐2 and NOX‐4 mRNA expression which was directly linked to its anthocyanin content (Petersen et al., 2018). Whilst we cannot determine the exact composition within EB that causes these effects it could likely be due to C3G phenolic metabolites which have demonstrated direct ROS scavenging and improved NO bioavailability (Edwards et al., 2015). Furthermore, to explain how EB scavenges ROS molecules we investigated Nrf2 expression (Anwar et al., 2014). The Nrf2 pathway is also linked to the inhibition of ROS generation via NADPH in EC (Petry et al., 2006). Interestingly only in the EB + TNF‐α, we observed an increase in Nrf2 expression (Figure 3). C3G has previously been demonstrated to induce Nrf2 expression and its antioxidant gene HO‐1 which suppressed TNF‐α induced nuclear translocation of NF‐κB p65 (Speciale et al., 2013).

As previously demonstrated, TNF‐α decreases eNOS expression and intracellular NO levels and markedly increases the expression of adhesion molecules (Obermaier et al., 2004). We detected no change in ICAM‐1 expression, although, the reduced VCAM‐1 expressed on cells limits monocyte capability to bind onto EC (Figure 6). Whilst both VCAM‐1 and ICAM‐1 are upregulated in atherosclerotic lesions, data indicates VCAM‐1 plays a predominant role in the initiation of atherosclerosis (Cybulsky et al., 2001). We, therefore, used an in vitro co‐culture model to investigate the functional capacity of EB to determine if it could reduce the adhesion of monocytes to EC. We found that EB reduced the adhesion of monocytes in the presence of TNF‐α (Figure 6). A similar anthocyanin‐grape‐rich extract could also prevent TNF‐α induced leukocyte adhesion to HUVECs in an epithelial‐endothelial co‐culture model (Kuntz et al., 2015). Blueberry metabolites attenuated endothelial inflammation in diabetic EC by suppressing monocyte binding and reducing IL‐8 and VCAM‐1 production (Cutler et al., 2018). However, in another study, despite reductions in IL‐6 very little difference in the inhibition of VCAM‐1 protein expression between the concentrations of the metabolites used to treat EC (Warner et al., 2017). Whilst mechanisms are still unclear, it is likely that EB which contains a mixture of metabolites, has some additive or synergistic activity in suppressing proinflammatory markers, including IL‐6 and MCP‐1, thus decreasing the crosstalk between EC and monocytes.

While this study successfully showed that EB improves different markers of ED the research conducted was limited to in vitro work. Moreover, we focused more on the synergistic bioactivity of EB on functional and mechanistic aspects of ED, than the specific components which might induce the observed effects. As briefly mentioned, we did detect C3G to be present in our extract (Figure S1). Recent studies have implied that C3G potentially exerts its functions primarily through its metabolites (Bharat et al., 2018), and more than 20 kinds of C3G‐metabolites have been identified in serum by a pharmacokinetics study in humans (Czank et al., 2013). Current findings demonstrate that phenolic metabolites derived from C3G are still present in human plasma for prolonged periods (< 48‐h), which demonstrates that monitoring EB functional capacity at these time points used within this study was worth being investigated (Czank et al., 2013). Despite the function and mechanisms of C3G‐metabolites are still not clear, metabolites do directly correlate with improved markers of endothelial function and should be explored further (Huang et al., 2021). Based on these findings and limitations, future studies should explore which EB anthocyanins and/or components are causing the described effects within this study to help for further application in vivo.

## 5 CONCLUSION

The main finding of this study demonstrates that EB improves molecular markers of ED linked to atherosclerosis. This suggests it could be used as a natural therapeutic supplement for improving endothelial function and protecting from the development of atherosclerosis. Future studies should explore if the findings of this study translate to an in vivo environment and determine a recommended concentration of EB extract that can be easily consumed.

## AUTHOR CONTRIBUTIONS

**Joseph Festa:** Conceptualization (supporting); data curation (equal); formal analysis (equal); methodology (supporting); writing – original draft (lead). **Aamir Hussain:** Data curation (equal); methodology (equal); writing – review and editing (equal). **Amon Hackney:** Methodology (supporting). **Unmesh Desai:** Methodology (supporting). **Tarsem S. Sahota:** Methodology (supporting). **Harprit Singh:** Conceptualization (equal); data curation (equal); formal analysis (equal); methodology (equal); supervision (supporting); writing – review and editing (equal). **Mariasole Da Boit:** Conceptualization (lead); data curation (equal); formal analysis (equal); methodology (equal); supervision (lead); writing – review and editing (equal).

## CONFLICT OF INTEREST STATEMENT

The authors declare they have no conflict of interest.

## Supporting information


Appendix S1.

## Data Availability

The data that support the findings of this study are available from the corresponding author upon reasonable request.
