# Supplementary material for: Elderberry extract improves molecular markers of endothelial dysfunction linked to atherosclerosis
Source: Food Sci Nutr. 2023 May 10;11(7):4047–59. doi: 10.1002/fsn3.3393 (PMC10345675; doi:10.1002/fsn3.3393)
Supplement: Supplementary file 1 — Appendix S1. [file FSN3-11-4047-s001.docx]

# **HPLC analysis - Anthocyanin extraction procedure**

The extraction of anthocyanins derived from Elderberry (EB) was done as mentioned previously (Youdim, Martin and Joseph, 2000). Briefly, EB samples were centrifuged at 10,000 rpm for 10 minutes and the supernatant collected. Consequently 100 μl 1N HCl was added to each pellet precipitate, vortexed vigorously and allowed to stand at 4°C for 20 min. The extraction procedure was repeated 2 times and the supernatants were combined for HPLC analysis.

**HPLC analysis**

Chromatographic separation was performed at 30°C using a HP Zorbax SB- C18 (stable bond) rapid resolution column (150 mm x 4.6 mm I.D.; particle size 3.5 μm, 80 Å) fitted with a Zorbax SB-C18 analytical guard column (12.5 mm x 4.6 mm; particle size 5 um, 300 Å). Aliquots (200 μl) of each extraction were injected for analysis using a Bio- analytical Systems Sample Sentinel autosampler maintained at 20°C (Model MF-9069) (West Lafayette, IN, USA). Normal phase separation of anthocyanins was achieved using binary gradient program Mobile phase A was 25 mM sodium acetate in water, mobile phase B was 25 mM sodium acetate in methanol, each adjusted to pH 1.5 with trichloroacetic acid. Initial starting conditions were 30% B, between 0–15 min % B increased from 30–35%, 15–20 min % B increased from 35–40%, 20–25 min % B increased from 40 –50%, at 25 min mobile phases were switched to original starting conditions (30% B) and held at this condition for 10 min prior to the next injection. The HPLC UV-Vis PDA sampler was set to a wavelength of 276 nm as determined from UV-Vis spectrophotometry (supplementary Figure 1A). The various compounds were characterized by chromatographic retention time comparisons against authentic standards of cyanidin 3-glucoside (C3G).

**Elderberry anthocyanin composition**

Figure 1B shows the chromatogram for cyanidin-3-glucoside (C3G) control with a peak at retention time 7.2 minutes. Figure 1C shows the chromatogram for EB with two main peaks at retention times of 6.56 and 7.06 minutes, the latter of which suggests the presence C3G in the EB.


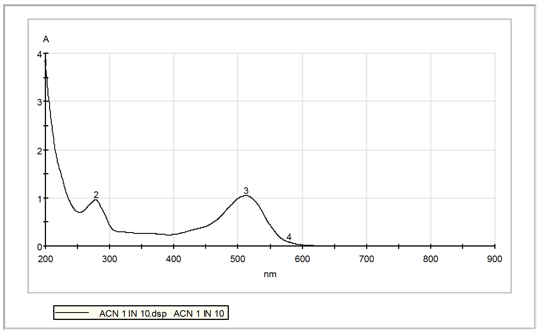

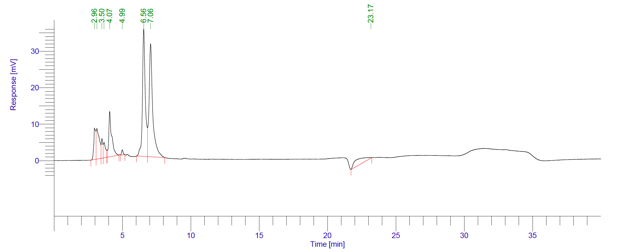

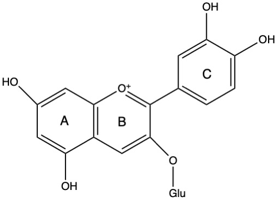

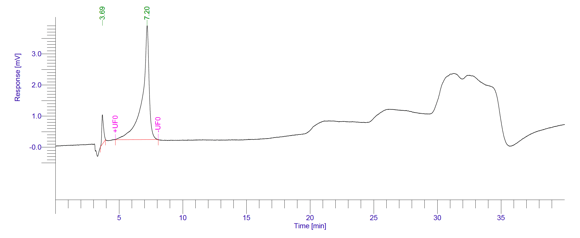


**(A)**

**(C)**

**(B)**

**Supplementary figure 1**. (A) HPLC UV-Vis PDA sampler was set to a wavelength of 276 nm as determined from UV-Vis spectrophotometry (B) Chromatograms showing cyanidin-3-glucoside control, (C) UV-vis spectrum of elderberry extract (EB) (50 ug/ml)
